# Antioxidant Defenses Predict Long-Term Survival in a Passerine Bird

**DOI:** 10.1371/journal.pone.0019593

**Published:** 2011-05-06

**Authors:** Nicola Saino, Manuela Caprioli, Maria Romano, Giuseppe Boncoraglio, Diego Rubolini, Roberto Ambrosini, Andrea Bonisoli-Alquati, Andrea Romano

**Affiliations:** 1 Dipartimento di Biologia, Università degli Studi di Milano, Milano, Italy; 2 Department of Zoology, University of Cambridge, Cambridge, United Kingdom; 3 Dipartimento di Biotecnologie e Bioscienze, Università di Milano-Bicocca, Milano, Italy; University of Turku, Finland

## Abstract

**Background:**

Normal and pathological processes entail the production of oxidative substances that can damage biological molecules and harm physiological functions. Organisms have evolved complex mechanisms of antioxidant defense, and any imbalance between oxidative challenge and antioxidant protection can depress fitness components and accelerate senescence. While the role of oxidative stress in pathogenesis and aging has been studied intensively in humans and model animal species under laboratory conditions, there is a dearth of knowledge on its role in shaping life-histories of animals under natural selection regimes. Yet, given the pervasive nature and likely fitness consequences of oxidative damage, it can be expected that the need to secure efficient antioxidant protection is powerful in molding the evolutionary ecology of animals. Here, we test whether overall antioxidant defense varies with age and predicts long-term survival, using a wild population of a migratory passerine bird, the barn swallow (*Hirundo rustica*), as a model.

**Methodology/Principal Findings:**

Plasma antioxidant capacity (AOC) of breeding individuals was measured using standard protocols and annual survival was monitored over five years (2006–2010) on a large sample of selection episodes. AOC did not covary with age in longitudinal analyses after discounting the effect of selection. AOC positively predicted annual survival independently of sex. Individuals were highly consistent in their relative levels of AOC, implying the existence of additive genetic variance and/or environmental (including early maternal) components consistently acting through their lives.

**Conclusions:**

Using longitudinal data we showed that high levels of antioxidant protection positively predict long-term survival in a wild animal population. Present results are therefore novel in disclosing a role for antioxidant protection in determining survival under natural conditions, strongly demanding for more longitudinal eco-physiological studies of life-histories in relation to oxidative stress in wild populations.

## Introduction

Normal physiological and immunological processes and diverse pathological conditions entail the production of substances with high oxidative potential, that can cause damage to the major classes of biological molecules (DNA, lipids and proteins), and interfere with a host of bodily functions [1]–[5]. The pervasive nature of oxidative challenge and the potentially severe negative consequences that the resulting oxidative damage has on fitness [1], [2], [6] have apparently driven the evolution of complex adaptations to prevent uncontrolled oxidation [3], [7], [8]. Antioxidant defenses typically involve integrated mechanisms that act at different levels of the oxidation cascade and may involve the intervention of endogenous mediators (e.g. enzymes) as well as exogenous elements and substances that are acquired with food (e.g. selenium, carotenoids and tocopherols) [7].

The fundamental biology of oxidative damage and the physiology of antioxidant defense have been at the focus of a large body of biomedical literature (e.g. [3]), but the role of prevention or repair of oxidative damage in shaping the evolutionary ecology and behavior of organisms in the wild has been fully appreciated only recently (see [8]–[11]). An imbalance between exposure to pro-oxidants and antioxidant defenses (i.e. ‘oxidative-stress’; see [4]), can result in severe depression of fitness traits such as fecundity, sperm quality and offspring quality via early maternal effects mediated by the egg, or increase the rate of physiological degeneration as individuals age [8], [12]–[14].

Because they rely on energy and resources (e.g. micronutrients) that may be limiting, antioxidant defenses may have to be traded against other physiological functions. On the other hand, the expression of main life-history traits, such as sexual behavior, reproduction and parental care can increase the threat of oxidative damage, and up-regulation of these functions may therefore have to be traded against the risk of incurring oxidative stress [8], [15], [16]. Because availability of resources, such as dietary antioxidants, is expected to vary with general ecological conditions, organisms should evolve life-history strategies that allow them to secure efficient antioxidant protection by optimizing allocation to antioxidant defense and competing functions.

The trade-off between the rate of functioning of the metabolic machinery and oxidative stress lies at the very core of some of the evolutionary theories of aging [1], [3], [17], [18], as individuals with a high production of oxidative substances are expected to be trading current investment in fitness traits against residual reproductive value. This ‘rate of living hypothesis’ [14], [19], [20] leads to the straightforward prediction that individuals that manage to maintain high levels of antioxidant defense enjoy larger survival.

While the physiological ecology literature on the short-term reciprocal links between antioxidants, oxidative stress and life-history has been flourishing in the last years (see [8], [10], [11], [21]–[24]), there is a dearth of studies linking oxidative stress to long-term survival in free-living vertebrates. This comes at no surprise, as analyses of survival in the wild are typically hampered by the difficulty of obtaining longitudinal information on vagile, long-lived organisms [25], and a physiological approach to the study of longevity has only recently been incorporated into ecological and evolutionary studies (e.g. [25], [26]).

In fact, to the best of our knowledge only one study could show an association between oxidative state and long-term survival in a free-living vertebrate. Breeding alpine swift (*Apus melba*) males that proved to be less susceptible to a specific type of oxidative damage were found to be more likely to return the following year to their local breeding population [27]. However, previous cross-sectional studies have investigated age-related variation in antioxidant capacity and markers of oxidative stress in free ranging populations of birds (e.g. [28], [29]). In the present study, we tested if plasma total antioxidant capacity (AOC) during the breeding season predicts annual survival. To this goal, we used a large data set on a colonial, aerially insectivorous migratory passerine bird, the barn swallow (*Hirundo rustica*), collected over five years. We predicted that the hazard of death between the breeding season of year *i* and year *i*+1 was reduced in individuals that had large AOC during the breeding season of year *i*. We had no specific ‘directional’ prediction on the patterns of sex-related variation in the effects of AOC on individuals of either sex, because the relative impact of sex-specific socio-sexual and reproductive activities on antioxidant defense is unknown.

An additional novel aspect of the present study is that it is based on longitudinal, rather than cross-sectional data and it therefore allowed to test for variation in AOC with age while taking into account the effect of selection. In fact, in cross-sectional analyses ontogenetic variation in any trait due to phenotypic plasticity cannot be distinguished from the effect of selection, and spurious associations between phenotype and age can arise at the population level if survival depends on phenotype [30]. Conversely, longitudinal analyses allow to uncover the actual association between phenotype and age, because the effect of selection can be discounted.

In the barn swallow, the length of the outermost tail feathers of males is a costly sexually selected trait, and individuals with longer outermost tail feathers have been repeatedly shown to be more viable and of higher phenotypic quality in the same population where the study was conducted, and elsewhere in Europe [31]–[33]. We therefore also tested whether any relationship between AOC and viability held after controlling for variation of male phenotypic quality as indicated by the size of their tail ornaments.

## Materials and Methods

Barn swallows were studied at three colonies. Data on AOC and survival refer to years 2006–2010, but additional information from 2002–2005 was also used to age the birds and to test age-related variation in AOC. Breeding adults were captured during the breeding period (1 May–20 June) by placing mist nets at the doors and windows of the rural buildings (mainly stables) where colonies were located, before dawn. This procedure is highly efficient in ensuring that very few breeding individuals (<5%) escape repeated capture attempts, as already shown in several previous studies (e.g. [34], [35]). Barn swallows in our study population, as well as in other European regions, show extremely high breeding philopatry, implying that adults that have bred in a given farm in year *i* do not move to another farm to breed in year *i*+1 (e.g. [31], [33], [34]). Because of high breeding philopatry and capture efficiency, individuals that were not found in any given year at the colony where they bred the previous year can be imputed as dead (see e.g. [31]). Of course, because barn swallows are migratory birds that leave the breeding colonies by July–August and migrate to overwinter south of the Sahara Desert before returning to the breeding colonies in March–May, we could not identify the exact time of death. We therefore imputed time of death as the time at last unsuccessful check for presence of that individual at its breeding colony in any given year *i*+1 and analyzed the hazard of death in relation to AOC in year *i* (see [36]; see also [27] for an analysis of annual survival). We considered last, rather than first check to allow for late arrival from migration.

At capture, standard measurements (see [37]), including the length of the outermost tail feathers, were taken and a small sample of blood (ca. 100 µl) was collected in capillary tubes after puncturing the brachial vein. Blood was kept in a refrigerated container while in the field and then taken to the lab. Plasma was separated by centrifugation within hours of collection and deep-frozen until biochemical analyses. Blood samples were not available from twelve individuals for one year in between years for which samples were available.

To analyze survival in relation to AOC we applied Cox proportional hazards regression models based on maximum partial likelihood estimation as implemented by PROC PHREG in SAS 9.2, using the “counting process” procedure [36]. Cox regression is widely used in survival analysis for several reasons, including that it does not require any a priori assumption about the probability distribution to model survival times and makes it easy to incorporate time-dependent covariates. Indeed, our sampling design involved that survival between year *i* and year *i*+1 for any given individual was modeled in relation to AOC recorded in any individual year *i* (i.e. AOC was a time-dependent predictor; see also above). Similarly, tail length was included in the models as a time-dependent covariate because tail feathers are molted annually, during winter, and tail length can thus vary between consecutive years [31]. Conversely, sex was modeled as a ‘time-independent’ covariate. Because age was not always known with certainty, data are ‘left-censored’. An event ( = death) was recorded whenever death could be imputed. To account for tied event times, we adopted the procedures implemented by PROC PHREG (TIES = EXACT), which is based on the probability of the union of the partial likelihoods for all possible orderings of tied events. Adoption of alternative procedures for handling tied events (see [36]) did not alter the quality of the results, and details of the results obtained with these alternative procedures will therefore not be shown. In the models, we allowed for stratification (see [36]) according to colony, in order to control for any variation in the shape of the hazard function between colonies, potentially due to spatial variation in ecological factors. Disregarding stratification, however, led to qualitatively similar results.

The analyses of survival were based on one estimate of AOC per individual and year obtained during the breeding season. Importantly, in a sample of 60 individuals from the same population that were sampled twice during the same breeding season, there was a strong positive correlation between the two measures of AOC (r = 0.52, P<0.001; repeatability computed according to Lessells and Boag (1987) [38]: R = 0.50, z = 3.44, P<0.001), implying that individuals that have relatively high AOC at any given stage of the breeding season tend to do so also at other stages. This is the case also for the concentration of individual antioxidants in the barn swallow (see [39]).

High repeatability (see [40]) justifies the use of one estimate of AOC per individual and year as a proxy for the relative AOC level during a given breeding season.

Age could confound the results of the analyses of survival in relation to AOC if survival rate covaries with age and AOC is also an age-dependent state variable. Age of individual swallows could be determined on a subset of observations by taking advantage of the high breeding philopatry of adults, because it implies that unmarked individuals that were captured in a given year *i* in a colony where the previous year all adults had been captured could be assumed to be 1-year old birds generated in another (or, more infrequently, in the same) colony during year *i*−1 (see [21], [34]). This procedure could obviously not be applied to breeding adults at the year of start of intensive ringing activity in individual farms, and this explains why the number of individuals (and of selection episodes = annual individuals' transitions) of known age is smaller than the total number of individuals (and selection episodes) for which survival information was available.

We tested for the potentially confounding effect of age in two ways. First, we checked whether AOC covaried with age. A simple test of the association between AOC and age as observed in the whole population would suffer from the potentially confounding effect of selection, because AOC may covary with age if individuals with large AOC are also better (or worse) at surviving. We therefore performed the analyses of AOC in relation to age by restricting the dataset to all and only the individuals that were recorded to have survived up to age 2 (i.e. excluding those that were recorded to have survived to age 1 or ≥3) (n = 116 observations). We then re-ran the analyses on the individuals that were recorded to have survived to age 3 (excluding those that survived to age ≤2 and those that survived to age ≥4) (n = 55). Finally the analysis was run on individuals that survived to age 4 (excluding those that survived to age ≤3 and those that survived to age ≥5) (n = 28). Sample size at older ages was too small to be amenable to analysis. These analyses were tackled by applying random intercept repeated-measures mixed models where individual was entered as a random effect and the linear and second order polynomial terms of age as predictors together with sex. Inclusion of age as a factor rather than as a continuous covariate did not alter the outcome of the analyses. In all analyses, degrees of freedom were estimated using the Kenward-Rogers method.

The second approach consisted of running a Cox regression model while including age as a covariate on the subset of data for which this information was available. Because this model led to results qualitatively similar to those on the larger, entire dataset, we will focus on the former analyses (see Results).

### Plasma antioxidant capacity

Because the different components of the antioxidant barrier do not necessarily act in an additive fashion, total antioxidant capacity is not simply an additive function of the concentration of individual antioxidants, and overall measures of antioxidant capacity are thus more representative of the redox status of an individual [8]. We therefore measured an index of overall plasma antioxidant capacity (AOC) using the OXY-Adsorbent test (Diacron, Grosseto, Italy) (see [41], [42] for application of the same protocol; see also [43] and references therein). This test performs a colorimetric determination of the capacity of the plasma antioxidant barrier to cope with oxidation by the hypochlorous acid (HClO). The plasma sample (5 µL) was diluted 1∶100 with distilled water. A 5 µL aliquot of the diluted plasma was added to 200 µl of a titred HClO solution. The solution was mixed and incubated for 10 min at 37°C. At the end of the incubation time, 5 µL of an alkyl-substituted aromatic amine solubilized in a chromogenic mixture, was added. This amine is oxidized by the residual HClO and transformed into a pink-colored derivative. The concentration of the colored complex is directly proportional to the HClO excess and inversely related to the antioxidant capacity of tested plasma. The intensity of the colored solution was measured at 492 nm using a photometer (Multiskan EX, Labsystem). One standard sample of known AOC and one blank sample (5 µL of distilled water) were also processed to be used as a control reference. Repeatability of AOC measures was tested on 62 samples that were assayed in duplicate, and was found to be large and highly significant (R = 0.65, z = 4.29, P<0.001).

In all analyses, statistics for AOC are given as mmol l^−1^ of HClO neutralised.

### Ethics statement

Upon capture, barn swallows were kept in cloth bags in a safe position not accessible to predators, as is standard practice in bird ringing studies. Blood samples were collected by puncturing the brachial vein and the puncturing site was accurately disinfected. All individuals were released as soon as possible, usually within 1 hour of capture. After being released, swallows behaved normally and observations at the nest on dozens of individuals confirmed that they resumed their normal breeding activities. The study was carried out under permission of the local authority (Regione Lombardia #M1.2005.0001845) responsible for authorizing animal studies in the wild.

## Results

The analyses of survival in relation to AOC were based on 427 selection episodes (225 for males, 202 for females) referring to 171 males and 154 females. Mean (SE) AOC for the 149 censored observations (i.e. individuals that were still alive the next year) was 175.64 (3.12), while the mean for the 278 measurements of AOC before recorded failure to return ( = death) was 167.88 (1.82) (see Methods for measurement units).

Interestingly, there was a highly significant repeatability of AOC values within individuals among years (R = 0.487, z = 6.79, P<0.001). Hence, not only individuals that had relatively high (or low) AOC at a certain time in any given breeding season tended to do so also at other times of the same breeding season (see Methods), but individuals with relatively high AOC in one year tended to do so also in other years.

**Figure 1:**
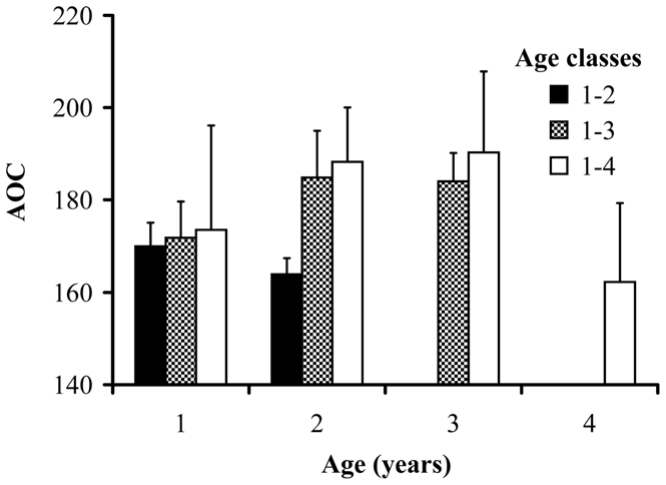
Survival of barn swallows in relation to antioxidant capacity (AOC; mmol l^−1^ of HClO neutralised). Survival functions were fitted to data stratified according to individuals being of low (< population mean −1 Standard Deviation), intermediate (comprised between mean −1 SD and mean +1 SD), or high (> mean+1 SD) AOC. This analysis differs from that presented in Table 1 and was performed to allow representation of the association between AOC and survival.

**Figure 2:**
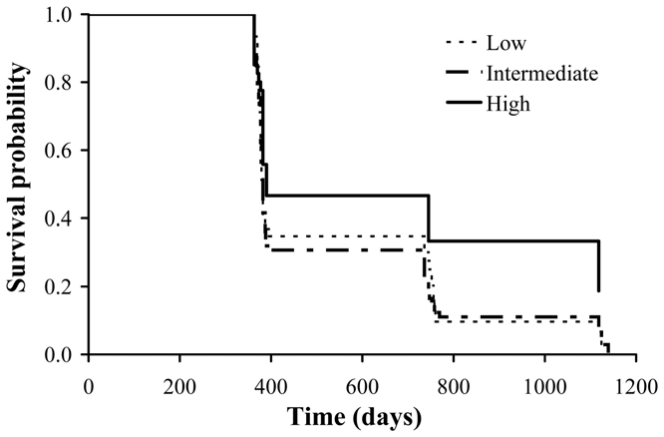
Antioxidant capacity (AOC; mmol l^−1^ of HClO neutralised) in relation to age. Mean (+ SE bar) AOC of individuals that were recorded to have survived up to 2 years (age class 1–2, n = 116), 3 years (1–3, n = 55), or 4 years (1–4, n = 28) (see Methods for details). For all age classes there was no significant variation of AOC with age in repeated-measures mixed models with individual as a random effect and age (covariate) and sex (factor) as fixed effects. When included in the model, the effect of the second order polynomial term of age was non-significant (see Results).

A simple Cox regression model with sex, AOC and their interaction as predictors revealed no significant effect of the interaction (χ^2^ = 0.07, df = 1, P = 0.789). After removal of the interaction effect, AOC was found to negatively predict the hazard of death (coefficient (SE) = −5.7 * 10^−3^ (2.0 * 10^−3^), χ^2^ = 8.32, df = 1, P = 0.004) (see Fig. 1). The associated hazard ratio estimate ( = 0.994) implies a 0.6% increase in survival per unit increase in AOC. Because the standard deviation of the AOC in the whole sample was 33.5, a 1 SD variation in AOC entailed a change in the hazard of death of ca. 20%. We tested whether age could be a confounding effect in the analyses of survival in relation to AOC while discounting the effect of selection, i.e. by analyzing in different repeated-measures mixed models the individuals that were recorded to have survived up to 2, 3 or 4 years (see Methods). In models including only the linear term, age did never significantly predict AOC (age 2: F_1,73.4_ = 1.48, P = 0.227; age 3: F_1,31.2_ = 0.27, P = 0.610, age 4: F_1,19.2_ = 0.80, P = 0.382; see Fig. 2). When included in the models, second order polynomial term of age also failed to predict AOC (age 3: F_1,26.3_ = 0.20, P = 0.655; age 4: F_1,16.1_ = 3.18, P = 0.093). Hence, there was no evidence that AOC covaried with age. Moreover, Cox analyses on a subset of data while including age as a covariate (see Methods) consistently showed that the effect of AOC on the hazard of death was highly significantly negative (coefficient (SE) = −6.8 * 10^−3^ (2.3 * 10^−3^), χ^2^ = 9.02, df = 1, P = 0.003). Hence, all the above analyses indicate that the effect of AOC on survival was not confounded by age effects. In the above analyses including age effects, when the interaction between age and AOC was included in the model it never attained statistical significance (details not shown).

In a model also including AOC and sex as predictors, length of the tail ornaments was found to negatively predict the hazard of death, while AOC retained its significant effect and was again associated to an hazard ratio of 0.994 (Table 1; see also above). Predicted survival for three classes of individuals (with low, intermediate or high AOC, respectively, see legend to Fig. 1 for definition of the classes) show that high AOC enhanced survival, whereas no appreciable differences existed between individuals with low or intermediate AOC. The two-ways interaction terms between AOC, sex and tail length did not predict the hazard of death both when they were entered one at a time or when they were entered simultaneously in the model (Table 1). Moreover, tail length did not predict AOC while controlling for the effect of sex (F_1,429_ = 0.56, P = 0.456) in a mixed model with individual as a grouping random effect, after excluding the non-significant sex by tail length interaction (F_1,448_ = 3.16, P = 0.076).

**Table 1:** Cox model of hazard of death in relation to sex, AOC and tail length.

	Coefficient (SE)	χ^2^	P	Hazard ratio
Sex*	−21.86 (17.58)	1.55	0.214	0.804
AOC*	−0.59 (0.20)	8.88	0.003	0.994
Tail lenght*	−1.69 (0.82)	4.24	0.040	0.983
Sex×AOC**	−3.46 (36.50)	0.01	0.924	/
Sex×Tail lenght*	−0.11 (1.86)	0.00	0.951	/
AOC×Tail lenght**	−0.27 (0.17)	0.03	0.873	/

Statistics for the main effects are obtained from a model which included no interactions. Statistics for individual interaction terms were obtained from different models including the main effects and the interaction term under scrutiny.

*: coefficients are multiplied by 10^2^;

**coefficients are multiplied by 10^4^.

## Discussion

We showed that a measure of antioxidant defense predicts annual survival of adult barn swallows. This is the first evidence for an association between viability and a general measure of the plasma antioxidant capacity in any vertebrate species under natural conditions, and complements the results of a previous study where annual survival of male alpine swifts was negatively predicted by susceptibility to oxidative damage of red blood cells [27]. A notable finding of this study was that AOC levels were repeatable within individuals not only within breeding seasons but also among years, suggesting that relative antioxidant capacity is consistently different among individuals throughout their lives.

These novel pieces of evidence corroborate the idea that defense from oxidative damage qualifies as a major factor influencing survival and thus potentially extending life-span, as envisaged by the free radical theory of aging [1], [2], [6]. Although controversy over the factual role of oxidative stress in affecting viability but also reproductive output and other life-history traits is still ongoing, a large body of evidence from captivity studies on ‘model organisms’, such as nematodes, insects and rodents indeed strongly advocates a major impact of detrimental oxidation processes (see [44], [45]). In addition, studies of captive zebra-finches (*Taeniopygia guttata*) showed that trade-offs among life-history traits could depend on conditions experienced early in life and be mediated by resistance to oxidative challenge [23]. Together with that by Bize and coworkers [27], though from a different perspective, the present study therefore adds to previous laboratory studies in demonstrating a role of protection from oxidative damage as determinant of individual survival prospects also under natural selection regimes.

The effect of AOC on viability was relatively strong, as an increase of 1 standard deviation in AOC was positively associated to a ca. 20% change in annual survival. A difference in the effect of AOC on viability between males and females could be expected, given the inherently different role of either sex in socio-sexual and reproductive functions, and thus oxidative challenge they entail (e.g. [46]), although the sign of this difference could not be predicted. On the one hand, females could be expected to benefit more from high AOC because they pay larger costs of reproduction both in general terms (i.e. energy for egg production) and in terms of allocation of antioxidants to the eggs and to the progeny via the eggs themselves [7]. On the other hand, males may be more exposed to oxidative stress because of their larger involvement in social interactions and sexual activities (e.g. singing, mate guarding, etc.). However, we found no evidence that the effect of AOC on survival was stronger in either sex. This may suggest that large AOC enhances resistance to oxidative damage mainly arising from activities that are common to either sex. Migration over long distances which is accomplished by both sexes with no evidence of spatial segregation (though with some temporal segregation; [47], [48]) is a main candidate context where high AOC may afford higher survival, independently of sex, because of the intense metabolic effort entailed by long-range migration [49]. Moreover, socially monogamous males and females may also similarly benefit from high AOC during post-natal offspring care, which is an energetically demanding period for both sexes, as parental duties are shared almost evenly between males and females [31].

The positive effect of AOC on viability was not confounded by age effects. Indeed, age did not predict AOC, and this was observed after discounting the effect of selection. This finding corroborates the inference of no variation of plasma antioxidant capacity with age recorded in a cross-sectional study of another passerine bird, the collared flycatcher (*Ficedula albicollis*) [29], although differs from the result of a study of the long-lived greater flamingo (*Phoenicopterus ruber roseus*), where resistance to oxidative stress was found to peak at intermediate ages (12–20 years) [28]. Moreover, the negative relationship between hazard of death and AOC emerged also in an analysis of a subset of individuals where we controlled for age effects. This is the first evidence based on longitudinal data from a natural population on the association between AOC and age. In the alpine swift, susceptibility to oxidative damage had opposite patterns of covariation with age in either sex. If that finding, which emerged from cross-sectional data, was not confounded by natural selection (see [27] for a discussion), the two studies combined suggest that individuals of either sex undergo differential age-related variation in oxidative stress, notwithstanding similar levels of antioxidant capacity. Obviously, this speculation must be taken with caution as the present study and that by Bize and coworkers [27] refer to distantly related species, although with similar trophic ecology.

A positive association of viability with AOC was found also while controlling for inter-individual variation in the expression of a sexual ornament. Long-tailed males are individuals of relatively high phenotypic quality and viability [31], [32]. Present results therefore indicate that the association of viability with physiological state was independent of any covariation between viability and other components of phenotypic quality, as reflected by tail length, which positively predicted viability, confirming previous studies on male barn swallows [31]. The effect of the sex by tail length interaction was non-significant, contrary to the expectation based on previous results and on sex-related variation in the function of tail length as a reliable indicator of phenotypic quality [31], [50]–[53]. Long-tailed females have been shown to arrive earlier from migration and to take shorter time to acquire a mate, thus suggesting that they may be of superior quality than short-tailed ones [31]. However, tail length did not predict viability of females [31]. The present study shows, however, that after controlling for antioxidant protection, the relationship between tail length and viability does not differ between sexes.

An open question remains over which mechanisms produce variation in AOC among individuals, and thus potentially affect viability via antioxidant protection. Individuals can differ in access to dietary antioxidants and this difference could be a persistent condition as implied by repeatability of AOC levels at different times in the breeding season but also among consecutive breeding seasons. In barn swallows, which defend nesting but not foraging territories, this might imply differences in aerial foraging ability and thus differences in supply of dietary antioxidants. Moreover, a positive association between AOC and viability may suggest that depletion of antioxidants in individuals with low AOC has not been sufficient to counter oxidative challenge and prevent the negative effects of oxidative damage on viability. In this perspective, low AOC may reflect large exposure to oxidative threat and thus potentially large oxidative damage. Immune processes may mediate the association between AOC and viability if large parasite burden entails depression of AOC to counter oxidative stress caused by immune response [4]. Variation in AOC may also reflect early ontogenetic processes caused by maternal effects [54]–[56]. There exists circumstantial evidence that egg antioxidants (e.g. carotenoids) have long-term ‘organizational’ effects on a host of traits, including absorption and use of antioxidants later in life [57]–[62]. Hence, variation in AOC may be the proximate reflection of maternal (and paternal) physiology and experience of environmental conditions, which have downstream consequences on offspring physiology. An alternative interpretation is that variance in AOC levels is mainly the reflection of additive genetic variation in any of the processes that affect AOC, including for example acquisition and absorption of antioxidants or susceptibility to oxidative damage [3], [7].

In conclusion, these novel findings based on longitudinal data show that long-term viability is predicted by protection against oxidative damage afforded by the multifaceted antioxidant system. These results complement previous evidence in demonstrating the potentially major role that resistance to oxidative stress has in determining viability under natural selection regimes. Moreover, for the first time this study demonstrates that antioxidant capacity does not covary with age, independently of natural selection. Several eco-physiological studies have addressed the links between oxidative stress and fitness-traits including fecundity and short-term viability. The potential role of oxidative stress in mediating life-history trade-offs strongly prompts for an extension of these studies to the longitudinal links between individual redox status and a major determinant of fitness such as long-term survival.
